# Thank You to Our GeoHealth 2023 Reviewers

**DOI:** 10.1029/2024GH001063

**Published:** 2024-04-17

**Authors:** Thanh H. Nguyen, Gabriel Filippelli, Susan C. Anenberg, Meredith Franklin, Tzung‐May Fu, Sagnik Dey, Karen Hudson‐Edwards, Sunny Jiang, Antarpreet Jutla, Yang Liu, Chiyuan Miao, Adina Paytan, Avner Vengosh

**Affiliations:** ^1^ University of Illinois Urbana‐Champaign Urbana IL USA; ^2^ Department of Earth and Environmental Sciences Indiana University Indianapolis IN USA; ^3^ Milken Institute School of Public Health George Washington University Washington DC USA; ^4^ University of Toronto Toronto ON Canada; ^5^ Southern University of Science and Technology Shenzhen China; ^6^ Indian Institute of Technology Research Executive Agency New Delhi India; ^7^ Camborne School of Mines and Environment and Sustainability Institute University of Exeter Exeter UK; ^8^ Samueli School of Engineering at UC Irvine Irvine CA USA; ^9^ Department of Civil and Environmental Engineering West Virginia University Morgantown WV USA; ^10^ Rollins School of Public Health Atlanta GA USA; ^11^ Faculty of Geographical Science Beijing Normal University Beijing China; ^12^ UC Santa Cruz Santa Cruz CA USA; ^13^ Nicholas School of the Environment Duke University Durham NC USA

**Keywords:** editorial, peer‐review

## Abstract

Peer‐review is the foundation and the safeguard of scientific research. Without the dedication of our reviewers, the journal would not have been successful. In 2023, 269 reviewers completed 434 reviews for the 174 manuscripts submitted to GeoHealth. Our reviewers are from all continents except Antarctica. Besides reviewers from North America, China, Europe, and China, we started to have reviewers from India, Latin America, and Africa. GeoHealth editorial board is committed to expanding the readership, authorship, and reviewership to other countries. If you have already reviewed for us, no matter where or who you are, we hope you and your colleagues will consider GeoHealth a home for your work. Below is the list of reviewers who completed more than two reviews or have outstanding quality reviews. Two of our reviewers are being nominated for AGU best reviewers awards.

## Key Points

- The editors thank the 2023 peer reviewers

Abhishek Anand

*Adam Tonks*

*Alan Kolok*

*Alexander More*

*Alexander Smith*

Alfonso Diz‐Lois Palomares

*Ali Shafqat Akanda*

*Aline Macedo de Oliveira*

*Allison Bredder*

*Amanda Hoffman‐Hall*

Amy Washbush

Andrea Sealy

Anita Santal

*Anna Luiza Bauer Canellas*

Anna Rosofsky

*Anni Yang*

*Anwesha Chakravarti*

*Arfan Arshad*

*Arthit Phosri*

Arun Sharma

*Azfar Hussain*

Barbara Dix

Ben Silver

Benjamin McMillan

Benjamin Zaitchik

*Bertrrand Fankam*

Bogdan Timar

Caitlin Gould

Cara Wychgram

*Carl Malings*

Carlos Bravo‐Vega

Carlos Mestanza

*Catherine Propper*

*Charlie Zhang*

*Cheikh Dione*

Chen Chen

Chen Wang

*Chenghao Wang*

*Chloe Brimicombe*

Christopher Weaver

Chuyuan Wang

*Craig Brown*

Dajun Zhao

Dan Brabander

Daniel Horton

Daniel Johnson

*Daniel Westervelt*

Dashan Wang

*David Gonzalez*

*David Kaiser*

David Schmale

David Stieb

Debajit Sarkar

*Debashis Nath*

*Dengjun Wang*

*Di Yang*

*Dianne Greenfield*

*Dongchuan Wang*

*Dong‐Wook Lee*

*E Harville*

Ediclê de Souza Fernandes Duarte

*Egide Kalisa*

Elizabeth Christenson‐Diver

*Elyse Caron‐Beaudoin*

*Ernest Asare*

Eungul Lee

Farhat Abbas

*Feng Chen*

Fengchao Liang

Fernando Garcia‐Menendez

Franklin Schwartz

Gaige Kerr

George Pomeroy

*Gerry Ryan*

*Grigore Herman*

Guanghui Huang

Gunnar Schade

*Haikun Wang*

Haitong Sun

*Haoling Chen*

Haris Majeed

*Hasnat Aslam*

Hayhow

Hazel Mooney

*Heather D'Angelo*

*Heidi Roop*

Hélder Lopes

Helena Chapman

*Hiroko Hort*

Hong Su

I Kioutsioukis

*Igor Dumic*

*Isheka Orr*

*Jagadeesh Puvvula*

Jamaludin Suhaila

*James Crooks*

Jane Baldwin

Jangho Lee

Janica Gordon

Jayajit Chakraborty

Jeffrey Wilson

Jennifer Harkness

Jennifer Horney

*Jenny Hand*

*Jeremy Hoffman*

*Jicheng Gong*

*Jie Ban*

*Joan Casey*

*Jon Rask*

*Joon Yul Choi*

Jordan Clark

Joseph McMillan

Joseph Servadio

*Joshua Cheng*

Julia Gohlke

Julie Snorek

Junran Li

*Kai Chen*

*Karen Holcomb*

*Karin Ardon‐Dryer*

Kashif Mehmood

*Kelly Smith*

Kelsey Gleason

*Kenan Li*

*Khanh Do*

*Khanneh Wadinga Fomba*

Kim Prisk

*Kirk Baker*

Kirk Hohsfield

*Kirpa Ram*

Korine Kolivras

Kristie Ebi

Kristopher Karnauskas

*Kwun Yip Fung*

Leticia Nogueira

Lianghua Zou

Lining Zhao

Liqin Wu

*Long Li*

Longxiang Li

Louie Rivers

Lovleen Gupta

Luciana Rizzo

*Luis Chaves*

Luís Novo

Lydia Jennings

*Lyssa Freese*

Madeline Schreiber

Manhattan Lebrun

Marc Debroe

Margaret Nguyen

*Margareta Pinter*

*Mariana Alifa*

Marianthi‐Anna Kioumourtzoglou

Marissa Childs

*Mark Smith*

*Marta Venier*

Martin Gabersek

Matthew Alvarado

Matthew Dellinger

Matthew Dietrich

*Matthew Ward*

Matthias Salomon

*Maya Chung*

Meghan Albritton

Meifang Li

Melissa Fiffer

*Melissa Lombard*

*Micah Hahn*

*Michael Hendryx*

*Michael Wimberly*

*Michelle Tomlinson*

Mina Aghaei

Ming Xu

*Minghao Qiu*

*Mingxing Chen*

Miranda Dally

Mitchell Manware

Mohamed Fethi Ben Hamouda

Mojisola Adeniyi

*Motomu Ibaraki*

Mouhamadou Bamba Sylla

*Muhammad Nawaz*

*Mulong Du*

*Muye Ru*

*Nancy Johnston*

Narendra Singh

*Naveen Joseph*

*Nelsha Athauda*

*Nicholas DeFelice*

Nicholas Mailloux

Nick Middleton

*Nidhi Singh*

*Nikhil P. Kothegal*

Noémie Letellier

Owen Duckworth

Patrick Kinney

*Peijun Shi*

Peng Zhou

*Pengfei Liu*

Peter Knappett

*Peter Macharia*

*Petre Bretcan*

Prashant Kumar

*Qiaohong Sun*

*Rachel Coyte*

Rachel Thomson

*Rachel Young*

Radley Horton

*Ramesh Dhiman*

*Rebecca Smith*

*Reshmita Nath*

Richard Remigio

Ritika Kapoor

*Ritu Parchure*

Robert Finkelman

Robin Rives

*Ryan Emanuel*

*Ryan Harp*

*Sagnik Dey*

*Sara Egendorf*

*Sarah Chambliss*

*Sarah Fortner*

*Sarah Paull*

*Sebastian Rowland*

Shakhawat Chowdhury

*Shannon Bartelt‐Hunt*

Shawn McElmurry

*Sheena Martenies*

Sheng Zheng

Shuai Shao

*Shubhayu Saha*

Simon Waddell

Slobodan Djordjevic

Sneha Gautam

*Solomon Bililign*

*Songshan Yue*

*Soumya Pal*

Sourangsu Chowdhury

*Stephanie Cleland*

Suli Huang

*Susan Anenberg*

T Agyekum

Tao Hu

*Tao Wen*

*Ted Smith*

Tewodros Godebo

Thomas Gill

*Thomas Heinze*

Tianheng Shu

*Valentina Gomez*

Vivek Ravichandran

*Wande Benka‐Coker*

*Wilson Salls*

*Xianghui Cao*

Xiaohui Wei

*Xiaoxue Ma*

Ximena Porcasi

Xuying Ma

*Yang Xie*

*Yanqing Xu*

Yao Qu

*Yiqun Ma*

*Yisi Liu*

Yuanjian Yang

*Yuewei Liu*

*Yuzhen Li*

Yuzhi Xi

*Zhenhua Zheng*

Zongwei Ma

